# Ultrathin Hybridized ZnOHF Nanowires with Enriched Oxygen Vacancies for High Selective CO_2_‑to-CO Electrocatalytic Conversion

**DOI:** 10.1021/acsami.5c10915

**Published:** 2025-09-08

**Authors:** Hsin-Chiao Wu, Chia-Chen Lin, Yu-Jen Chou, Shu-Yu Lee, Shih-Han Wang, Tsan-Yao Chen, Ta-Chung Liu

**Affiliations:** † Department of Biomedical Engineering, 34914 National Yang Ming Chiao Tung University, Taipei 112, Taiwan; ‡ Department of Mechanical Engineering, 34878 National Taiwan University of Science and Technology, Taipei 106, Taiwan; § Department of Engineering and System Science, 34881 National Tsing Hua University, Hsinchu 300, Taiwan

**Keywords:** CO_2_RR, hybridized, heterointerface, oxygen vacancy, nanowire, zinc, fluorine

## Abstract

Electrochemical CO_2_ reduction reaction (CO_2_RR) has emerged as a key negative-emission technology, yet its industrial adoption hinges on cathode catalysts that deliver high selectivity and production rates at low cost. Herein, we reported a facile hydrothermal route to synthesize different scales of ZnOHF ultrathin nanowires with hybridized ZnO/ZnOHF heterointerfaces, where the 40 nm variant (NW_40_-ZnOHF) showed a high FE of 93 % and a *j*_CO_ of −17.2 mA/cm^2^ at −1.0 V vs RHE. The excellent intrinsic electrocatalytic properties of NW_40_-ZnOHF were verified, presumably owing to its high-level surface concentration of oxygen vacancies preexisted in the pristine structure and formed during CO_2_RR. This work highlights the significance of the synergistic effect of heterointerface and fluorine incorporation engineering for guiding the optimized design of low-cost electrocatalysts for CO_2_RR technology.

## Graphical abstract

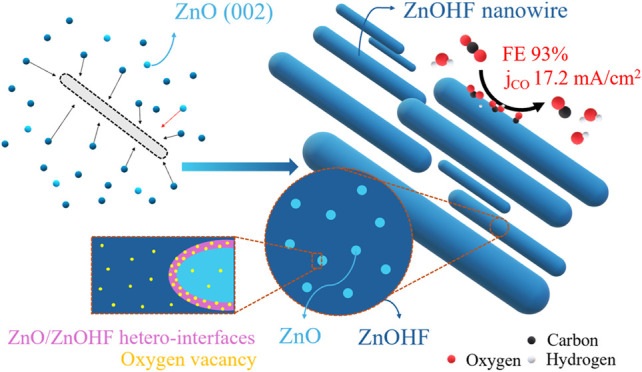

## 1 Introduction

Electrochemical carbon dioxide reduction reaction technology (CO_2_RR) has emerged as a promising route to tackle the rising CO_2_ concentration since it can be driven by renewable energy sources, producing high value-added chemicals and fuels under ambient temperature and pressure, with the implementation of this process largely relying on the development of low-cost and efficient electrocatalysts.^1–3^ The cathode electrocatalyst in a CO_2_RR system is of particular importance, as it governs the range of products, production rate, and overall product selectivity.^4,5^ Traditionally, precious metal catalysts, which offer high electrical conductivity and stable CO_2_ adsorption-free energy, have been widely studied and applied.^6,7^ However, industrial-scale CO_2_RR demands cost-effective solutions for economic feasibility,^8^ prompting extensive interest in developing lower-cost electrocatalysts such as Pb for formic acid production,^9^ Mn for methane,^10^ and Zn for CO.^2^

CO_2_RR-to-CO is often viewed as the most commercially promising pathway, primarily because it requires only two electron transfers (as opposed to eight for CH_4_ production) and can generally achieve higher Faradaic efficiencies (FE) and massive reaction currents.^8,11^ In such CO_2_RR-to-CO cascade reaction, a mechanism for CO_2_RR involves two proton-coupled electron transfers (PCET) as following steps^12^

$${\;}^{*}+{\mathrm{CO}}_{2}\to {\;}^{*}{\mathrm{CO}}_{2}$$

CO2*+e−+H+→COOH*

COOH*+e−+H+→CO*+H2O

CO*→*+CO

Among the diverse CO_2_RR-to-CO electrocatalysts, Zn-based materials stand out as a cost-effective option, and various morphologies have been extensively investigated (Table 1), including nanoparticles, nanorods, nanosheets, and so on.^13–22^ They are believed to yield high CO_2_RR selectivity by their intrinsic high affinity to CO_2_ and relatively poor affinity to CO and can be manipulated by controlling specific surface crystal facets and defects to reduce the free energy of *COOH or *CO intermediates, thus accelerating the PCET step and favoring CO_2_RR over the hydrogen evolution reaction (HER). Recently, heterointerface engineering offered a versatile strategy for CO_2_RR by introducing a variety of active sites that adjust the electron configurations to lower the free energies of key intermediates and thereby enhance product selectivity.^23^ For instance, Tang et al. anchored Ag nanoparticles onto oxygen-vacancy-rich CeO_2_ nanorods.^24^ The resulting Ag/O_V_-CeO_2_ heterointerface optimized the electronic structure of Ag while simultaneously creating additional *COOH adsorption sites on the nanorods, strengthening *COOH adsorption without hindering *CO desorption. Wang et al. grew ZnO*_x_* nanoparticles directly on Cu foil,^25^ creating a Cu/ZnO*_x_* heterointerface in which the localized ZnO_*x*_ domains stabilize Cu^2+^ species. Density functional theory (DFT) calculations revealed that Cu^2+^ sites provide the most favorable landscape for all intermediates involved in CO_2_RR to CH_4_. Together, these advances highlight the crucial roles of heterointerface construction in enhancing CO_2_RR performance.

**Table 1:** Comparisons of Some Systems Using H-Cell

material	electrolyzer	electrolyte	FE(%)	potential	*j*_CO_
Zn nanoplate (H–Zn–NPs)^13^	H cell	0.1 M KHCO_3_	94.2	–0.96 V vs RHE	6.78 mA/cm^2^
porous Zn (P–Zn)^14^	H cell	0.1 M KHCO_3_	94.4	–0.95 V vs RHE	25.5 mA/cm^2^
Zn nanosheets (MZN)^15^	H cell	0.5 M KCl	86.6	–1.75 V vs Ag/AgCl	5 mA/cm^2^
N-doped ZnO nanorods^16^	H cell	0.5 M KHCO_3_	76	–0.7 V vs RHE	3.5 mA/cm^2^
ZnO nanosheet (ZnO NS)^17^	H cell	0.1 M KHCO_3_	84	–1.2 V vs RHE	8.4 mA/cm^2^
(101) ZnO nanorod^18^	H cell	0.1 M KHCO_3_	91.4	–0.9 V vs RHE	∼12 mA/cm^2^
ZnO supported Ag^19^	H cell	0.5 M KCl	95	–1.1 V vs RHE	21.38 mA/cm^2^
Zn/NC NSs^60^	H cell	0.5 M KHCO_3_	98.2	–0.5 V vs RHE	2.25 mA/cm^2^
Zn_1_NC^61^	H cell	1 M KHCO_3_	98.8	–0.4 V vs RHE	10 mA/cm^2^
Zn-MOFs (FJU-127-CH)^63^	H cell	0.5 M KHCO_3_	90.2 (HCOOH)	–1.57 V vs RHE	24 mA/cm^2^ (for *j*_HCOOH_)
Zn-MOFs (2N–Zn-MOF)^62^	H cell	0.5 M KHCO_3_	31 (CO + CH_4_)	–0.45 V vs RHE	2.91 mA/cm^2^ (for *j*_CO_ + *j*_CH4_)
NW_40_-ZnOHF (this work)	H cell	0.1 M KCl	93	–1.0 V vs RHE	17.2 mA/cm^2^

According to our previous work, the ZnF_2_ nanogel achieved a faradaic efficiency of up to 82% under low overpotentials over ZnO nanoparticles by lowering the formation energy of *COOH intermediate instead of undergoing hydrogen evolution, verified by DFT calculation.^44^ In addition, abundant studies have demonstrated that electrocatalysts modified with fluorine exhibit enhanced CO_2_RR performance. For example, Zhou et al. employed CF_4_ plasma to fluorinate a copper surface, thereby reducing the size of copper particles and generating Cu–F bonds.^26^ These modifications stabilized Cu^+^ species, which in turn improved the adsorption of *CO and *CHO intermediates and enabled an 81.8% FE for C_2_^+^ products at −0.56 V vs RHE in a flow cell. Wei et al. used thermal copyrolysis of PTFE and carbon sources to fabricate fluorine-doped cage-like porous carbon.^27^ Finite element simulations revealed that K^+^ ions accumulate near the F–CPC surface, reducing the free energies of *COOH and *CO and achieving an FE of 88.3% for CO at −1.0 V vs RHE in an H-cell. Similarly, Han et al. incorporated fluorine atoms into a single-atom Ni catalyst on fluorine/N codoped carbon via PTFE pyrolysis.^28^ DFT calculations indicated a substantial *U*_L_(CO_2_) – *U*_L_(H_2_), in which *U*_L_ = −Δ*G*_0_/*e*. This confirms that Ni-SAs@FNC lowers the adsorption energy of *COOH and weakens *H affinity, enabling a 95% FE for CO at −0.77 V vs RHE.

Herein, driven by the F doping and heterointerface hybridized engineering, we reported ultrathin ZnOHF nanowires with diameters ranging from 20 to 75 nm featuring a ZnO(002)/ZnOHF hybridized structure. The ZnOHF/ZnO heterointerfaces were formed via the competition between OH^–^ and F^–^ reacts with Zn^2+^ under basic reduction environment. In addition, multiple structural investigations have verified the existing oxygen vacancies in the pristine structure. The results showed NW_40_-ZnOHF achieved a 93% FE at −1.0 V vs RHE (18.5 mA/cm^2^) in an H-cell. The enriched oxygen vacancies preexisted in the pristine structure and formed during CO_2_RR are considered critical indicators for enhancing CO selectivity.

## 2 Experimental Section

### 2.1 Materials and Reagents

Anhydrous zinc fluoride (ZnF_2_) was purchased from Alfa Aesar. Hexamethylenetetramine puriss. (HMT) was obtained from Riedel-de Haën. Potassium chloride (KCl), potassium hydroxide (KOH), and potassium bicarbonate (KHCO_3_) were sourced from Sigma-Aldrich. 95% sulfuric acid (H_2_SO_4_) and 30% hydrogen peroxide (H_2_O_2_) were acquired from Honeywell Fluka. 99.8% isopropyl alcohol (IPA) was bought from Emperor Chemical CO. Ltd. Gas diffusion electrode (GDL-AvCarb GDS5130, GDE) and Sustainion were supplied from Dioxide Materials. Nafion 115 was purchased from Chemours. Ag/AgCl reference electrode was obtained from ALS Co., Ltd. Japan. Platinum sheet (Pt) and Titanium (Ti) foam were sourced from Imgjing. 36.5–38% hydrogen chloride (HCl) was acquired from J.T.Baker. Dihydrogen hexachloroplatinate (H_2_PtCl_6_·*x*H_2_O) was bought from UniRegion Bio-Tech. Carbon dioxide (CO_2_), Helium (He), and Nitrogen (N_2_) were supplied from C. C. Gaseous Corporation. Methanol (CH_3_OH, MeOH) and acetone were purchased from Grand Chemical CO. Ltd.

### 2.2 Synthesis of Pristine ZnOHF and Ultrathin ZnOHF Nanowires

A 0.1 wt % ZnF_2_ dispersion was prepared in H_2_O, followed by the addition of various amounts of HMT. Specifically, the molar ratios of ZnF_2_ to HMT in the dispersion were set to 1:0, 1:1, 1:2, and 1:4. The mixture was stirred at 80 °C for 3 h, and the resulting precipitate was collected by centrifugation. After drying in an oven, the samples were labeled as P-ZnOHF, NW_75_–ZnOHF, NW_40_-ZnOHF, and NW_20_-ZnOHF, respectively. Prior to the cathode preparation, 15 mL of all sample dispersions in MeOH were prepared. The dispersions were then ultrasonicated for 1 h to ensure uniform mixing.

### 2.3 Cathode Preparation

An ultrasonic spray coating system was used to deposit 0.5 mg/cm^2^ of P-ZnOHF, NW_75_–ZnOHF, NW_40_-ZnOHF, and NW_20_-ZnOHF on 1 cm × 2 cm GDE, respectively. A peristaltic pump delivered 1 wt % P-ZnOHF, NW_75_–ZnOHF, NW_40_-ZnOHF, and NW_20_-ZnOHF in MeOH to the ultrasonic spray system at 0.25 mL/min. Then, the solution was sintered onto the GDE heated at 170 °C by a hot plate with an ultrasonic power of 23 V and 0.7 A until 0.5 mg/cm^2^ of P-ZnOHF, NW_75_–ZnOHF, NW_40_-ZnOHF, and NW_20_-ZnOHF were coated. After spraying, the coated GDE cathode was placed in an oven at 50 °C overnight to remove residual solvents.

### 2.4 Electrochemical Measurements

Unless otherwise stated, all electrochemical measurements were conducted in the H-cell. A Nafion 115 membrane was used to separate the cathode and anode. A 5 cm^2^ platinum foil served as the anode, and 0.1 M KCl solution was used as the electrolyte. An Ag/AgCl electrode saturated with 3 M KCl was used as a reference electrode. The catholyte was purged with pure CO_2_ or N_2_ at a rate of 50 mL/min for 1 h before conducting linear sweep voltammetry (LSV), cyclic voltammetry, and chronoamperometry. The following equation converted the reversible electrode potential (RHE)

E(RHE)=E(Ag/AgCl)+0.198+0.0591×pH+IR

The current interruption method (CI method) was used to compensate for the ohmic drop correction by determining catholyte resistance.^58,59^ Electrochemical tests were carried out with an OctoStat200 R31660 potentiostat (IVIUM Technologies, Nederland) controlled by Iviumsoft software. The pH value was measured with a HI2002–01 pH meter (HANNA Instruments).

### 2.5 Product Evaluation

The gaseous products from the cathode compartment were analyzed using an HP Agilent 6890 Plus GC (G1530A). The GC was equipped with a ShinCarbon ST, 100/120 mesh, 2 m, 1/16 in. OD, 1.0 mm ID (cat.#19808) column operated with thermal conductivity and flame ionization detectors. All liquid products were analyzed by JEOL NMR500 (ECZ500R/S1), and their yields were negligible. Gaseous products were injected into the GC by extracting 1 mL of gas from the cathode compartment after 20 min of sealed reaction. Helium was employed as a carrier gas for CO analysis, while nitrogen served as a carrier gas for H_2_ detection. The partial current (*j*_P_) and FE were obtained from the following equation

$$j_{P}=\frac{\mathrm{FzPV}}{\mathrm{tRTX}},\mathrm{FE}=\frac{j_{P}}{j}$$

where *t* is the reaction time; *R* is the ideal gas constant; *T* is the cathodic temperature; *F* is Faraday’s constant; *z* is the number of electrons transferred in the CO_2_RR equation; *P* is the cathodic pressure; *V* is the measured volume; *X* is the sample volume fraction; *j* is the total current.

### 2.6 Materials Characterization

The morphologies and microstructures of the four ZnOHF samples were examined using a JEOL JSM-7600F field emission scanning electron microscope (SEM) and a FEI Tecnai G2 F-20 S-TWIN transmission electron microscope, both equipped with an energy dispersive spectroscopy (EDS) detector. X-ray diffraction (XRD) analyses were performed on a Bruker AXS GmbH D8 Advance Powder X-ray Diffractometer using a Cu Kα beam, with the ZnOHF nanowires prepared in powder form. X-ray photoelectron spectroscopy (XPS) spectra were recorded using a Thermo Scientific Theta Probe system, with the ZnOHF samples coated onto a gas diffusion electrode (GDE). Raman spectra were obtained using a Horiba FHR 640 Symphony Raman spectrometer (LN2) equipped with a 532 nm laser operating at less than 25 mW. Electron paramagnetic resonance (EPR) spectra were recorded using a Bruker EPR- plus 2017, with the ZnOHF samples coated onto a gas diffusion electrode (GDE). In-situ XAS spectra at Zn K-edge was measured in transmission mode at beamlines BL13-B1 of NSRRC, Taiwan. The acquired EXAFS data were processed with the Athena software package, with the spectra weighted by *k* ^3^ to enhance oscillation features in *k*-space. Subsequent Fourier transform analysis was applied to convert the data into R-space, facilitating the investigation of bond length variations across different sample conditions.

## 3 Results and Discussion

### 3.1 Morphology of Ultrathin ZnOHF Nanowires

Figure 1a–c displayed the SEM images of the ultrathin ZnOHF nanowires synthesized under varying reactant ratios of ZnF_2_ to HMT (1:1, 1:2, and 1:4, respectively), which were designated as NW_75_–ZnOHF, NW_40_-ZnOHF, and NW_20_-ZnOHF, respectively. The formation of these nanowires could be explained by the following reactions, in which HMT or NH_4_^+^ ions would adsorb onto the nanowire sidewalls and thereby guide growth preferentially along a single crystallographic axis^29–31^

$${\mathrm{ZnF}}_{2}↔Zn^{2+}+2F^{-}$$

$$\mathrm{HMT}+6H_{2}O↔{4\mathrm{NH}}_{3}+6\mathrm{HCHO}$$

$${\mathrm{NH}}_{3}+H_{2}O↔{{\mathrm{NH}}_{4}}^{+}+{\mathrm{OH}}^{-}$$

$$Zn^{2+}+2{\mathrm{OH}}^{-}↔\mathrm{Zn}{\left(\mathrm{OH}\right)}_{2}↔\mathrm{ZnO}+H_{2}O$$

$$Zn^{2+}+F^{-}+{\mathrm{OH}}^{-}↔\mathrm{ZnOHF}$$

Note that due to the competitive reaction between OH^–^ and F^–^ with Zn^2+^, all the ZnOHF samples could exhibit both ZnO and ZnOHF, showing a possible hybridized structure. The resulting geometric irregularly shaped P-ZnOHF (Figure S1) highlighted the importance of HMT in directing the nanowire growth of ZnOHF. The effect of different HMT addition ratios impacted the size (wire diameter) and uniformity of ZnOHF nanowires. For each sample, the calculation of wire diameter was defined by collecting 30 random measurements from multiple SEM images to determine the average nanowire diameter and corresponding standard deviation. As shown in Figure 1d–f, the diameters of NW_75_–ZnOHF, NW_40_-ZnOHF, and NW_20_-ZnOHF were 75 ± 24.54, 40 ± 11.32, and 20 ± 3.25 nm, respectively. With increasing HMT content, the ZnOHF diameter decreased, and the uniformity of the rod size improved, demonstrating that HMT played a significant role in controlling the growth of thinner ZnOHF nanowires.

**Figure 1:**
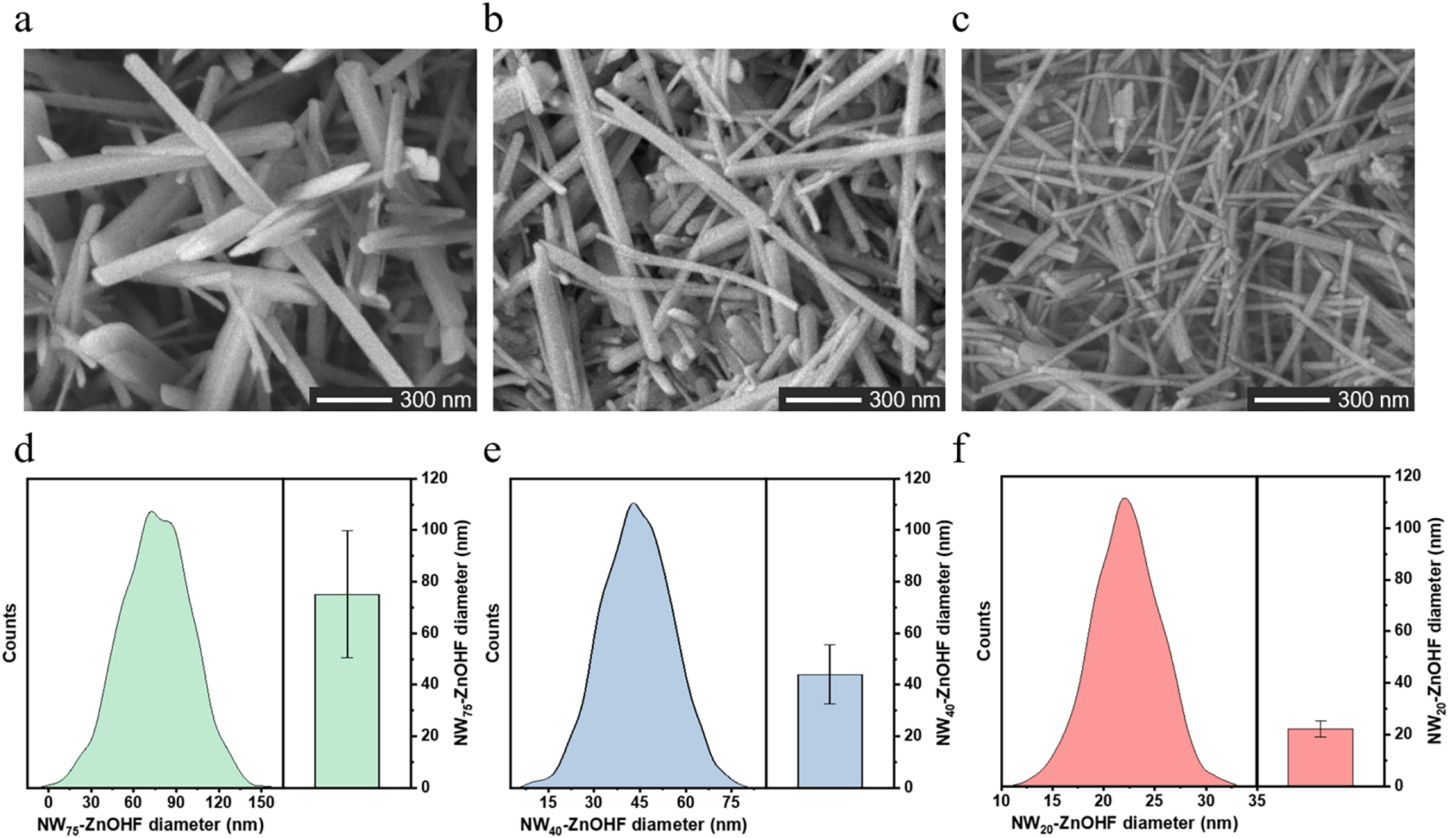
SEM images of (a) NW_75_–ZnOHF, (b) NW_40_-ZnOHF, and (c) NW_20_-ZnOHF. Nanowire diameter distribution of (d) NW_75_–ZnOHF, (e) NW_40_-ZnOHF, and (f) NW_20_-ZnOHF.

### 3.2 CO_2_RR Performance

We investigated the CO_2_RR performance of P-ZnOHF, NW_75_–ZnOHF, NW_40_-ZnOHF, and NW_20_-ZnOHF in H-cell. In Figures 2a and S2a, all samples achieved high selectivity of over 80% FE_CO_ at moderate potential, a value higher than ZnO nanoparticles (highest 71% FE at −1.1 V vs RHE) and ZnF_2_ nanogel (highest 82% FE at −1.05 vs RHE) according to our previous work.^44^ This might be attributed to the incorporation of F and the enhanced active catalytic sites in the ZnO/ZnOHF hybridized structure.^32^ The FE_CO_ for CO_2_RR from −0.7 to −1.4 V vs RHE followed a volcano-shaped trend for all samples, similar to most zinc-based CO_2_RR catalysts.^33,34^ This behavior is likely attributed to the thermodynamic differences in reduction potentials between HER and CO_2_RR, as well as mass transport limitations for CO_2_ at higher overpotentials.^35^ However, NW_40_-ZnOHF achieved its highest FE_CO_ at −1.0 V vs RHE, which was more positive than the potentials at which P-ZnOHF, NW_75_–ZnOHF, and NW_20_-ZnOHF reach their maximum FE.^36–38^ Moreover, 93% FE_CO_ of NW_40_-ZnOHF outperformed other electrocatalysts across the entire potential range, underscoring its superior CO_2_RR catalytic activity. Figures 2b and S2b,c presented the partial current density for CO, the total current density, and the partial current density for HER, respectively. At −1.0 V, the current density of NW_40_-ZnOHF exceeded the corresponding current densities of P-ZnOHF, NW_75_–ZnOHF, and NW_20_-ZnOHF at their maximum FE_CO_ (Figure 2b). This indicated that NW_40_-ZnOHF could deliver a higher CO_2_RR-to-CO production rate with reduced energy consumption. Table 1 summarizes the electrocatalytic properties of NW_40_-ZnOHF alongside those of previously reported Zn-based systems.

**Figure 2:**
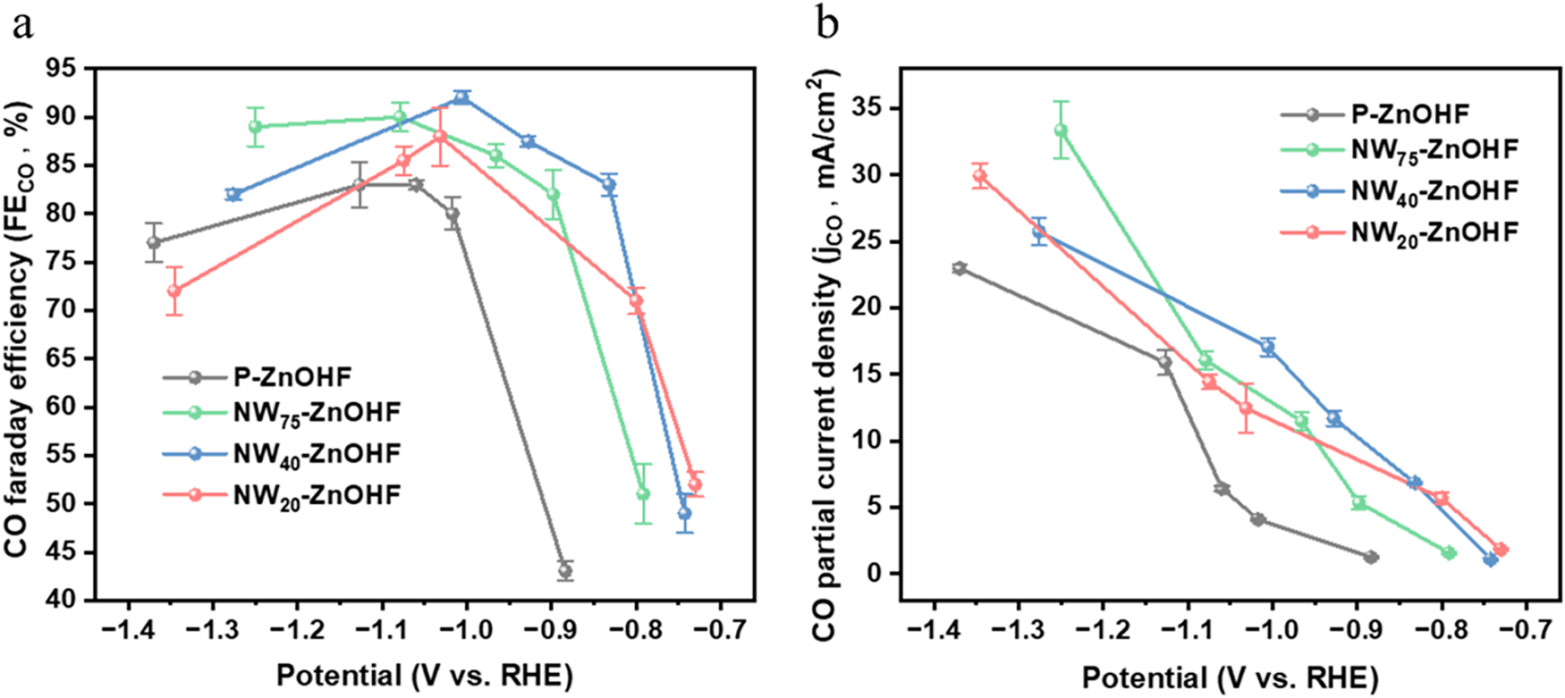
(a) FE_CO_ and (b) *j*_CO_ plots of P-ZnOHF, NW_75_–ZnOHF, NW_40_-ZnOHF, and NW_20_-ZnOHF in H-cells.

### 3.3 Electrode Kinetics for Electrocatalytic Performance

To further investigate the electrocatalytic behaviors of P-ZnOHF, NW_75_–ZnOHF, NW_40_-ZnOHF, and NW_20_-ZnOHF, we performed Tafel and ECSA analyses to identify the possible mechanisms from views of electrode kinetics and thermodynamics. LSV scans were conducted at 50 mV/s for all four electrocatalysts under N_2_- and CO_2_-saturated conditions, respectively, as shown in Figure S3a,b. By comparing the LSV curves for both gas atmospheres at higher overpotentials, only NW_40_-ZnOHF exhibited a significantly higher current density under CO_2_-saturated conditions than under N_2_-saturated conditions, suggesting superior CO_2_RR selectivity and production rates. Note that under an N_2_ atmosphere, HER is the predominant reaction. As illustrated in Figure 3a,3b, all the ZnOHF nanowires exhibited a more negative onset overpotential with their own Tafel slopes in the CO_2_-saturated condition compared to the N_2_-saturated condition, reflecting a typical HER poisoning effect.^39^ Since CO_2_RR and HER often share the same active sites, CO_2_ adsorption on the catalyst hinders HER. This observation clarified that P-ZnOHF, NW_75_–ZnOHF, NW_40_-ZnOHF, and NW_20_-ZnOHF showed high affinity to CO_2_ adsorption, and it further suggested that all four materials could achieve high selectivity of over 80% FE at moderate potentials (Figure 2a).

**Figure 3:**
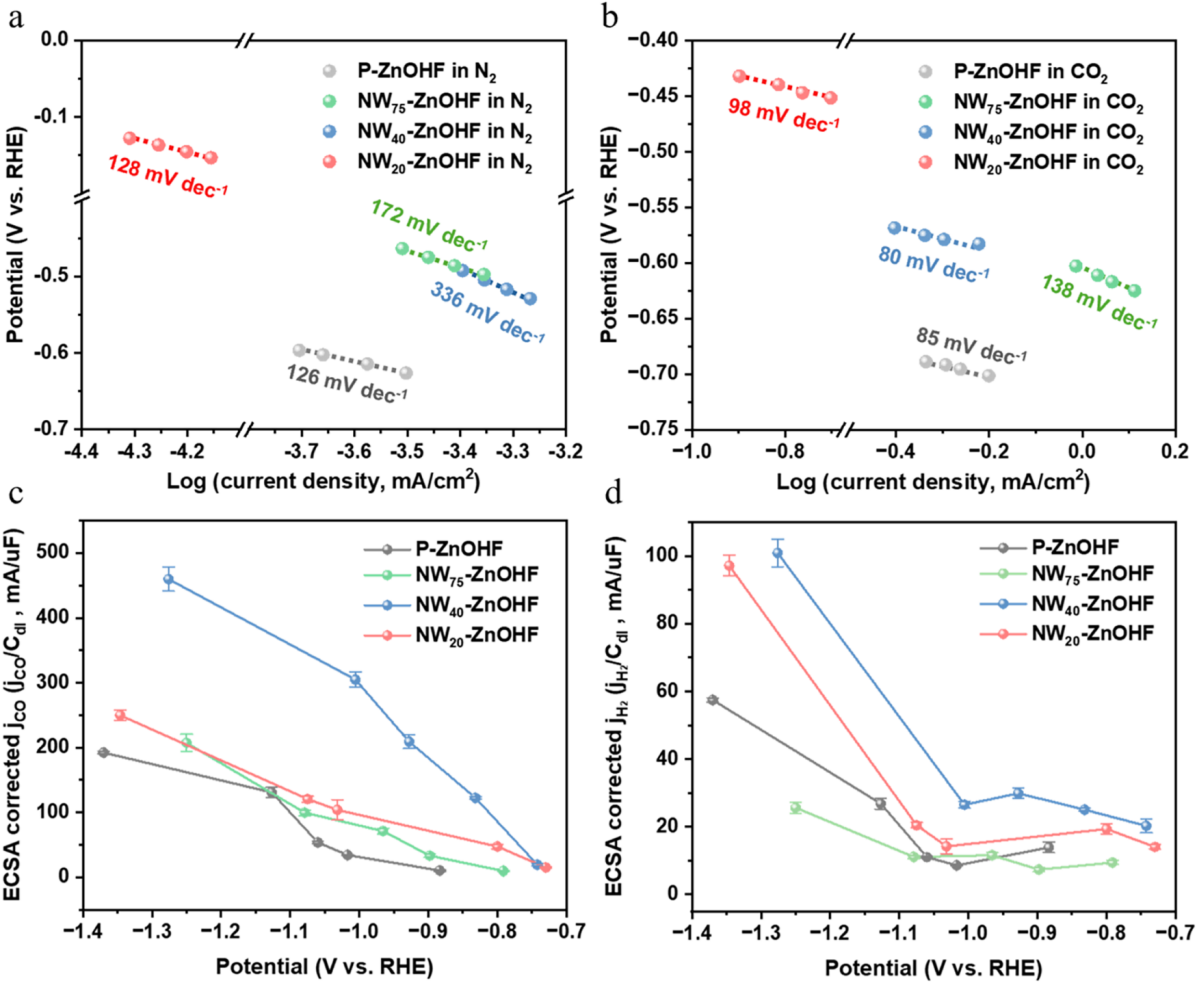
Tafel slope of P-ZnOHF, NW_75_–ZnOHF, NW_40_-ZnOHF, and NW_20_-ZnOHF under (a) N_2_- and (b) CO_2_-saturated conditions. The ECSA corrected (c) *j*_CO_ and (d) *j*_H2_ of P-ZnOHF, NW_75_–ZnOHF, NW_40_-ZnOHF, and NW_20_-ZnOHF.

Figure 3a revealed that under N_2_ conditions, the Tafel slopes for P-ZnOHF and NW_20_-ZnOHF were close to 120 mV/dec, indicating that their rate-determining step (RDS) for HER was the Volmer step.^40^ In contrast, NW_75_–ZnOHF and NW_40_-ZnOHF displayed Tafel slopes that are considerably greater than 120 mV/dec, with NW_40_-ZnOHF reaching 336 mV/dec, which deviated the most from 120 mV/dec These implied that NW_75_–ZnOHF and NW_40_-ZnOHF had more pronounced kinetic constraints on HER, with NW_40_-ZnOHF encountering the greatest limitation. In Figure 3b, the Tafel slopes of P-ZnOHF, NW_75_–ZnOHF, NW_40_-ZnOHF, and NW_20_-ZnOHF under CO_2_-saturated conditions were all lower than those under N_2_-saturated conditions, with NW_40_-ZnOHF showing the largest decrease from 336 mV/dec in N_2_ to 80 mV/dec in CO_2_. Note that Tafel slopes of approximately 118, 59, and 270 mV dec^–1^ correspond to the RDS of the first PCET step, the second PCET step, and an adsorption/desorption step, respectively, according to previous research.^41,42^ P-ZnOHF, NW_20_-ZnOHF, and NW_40_-ZnOHF exhibit Tafel slopes of 59–118 mV/dec The interfacial electric field, intermediate coverage-potential coupling and mass transport effects stabilize the *CO_2_ transition state and raises the effective transfer coefficient to 0.7–0.8, thereby reducing the theoretical Tafel slope for the first PCET step from 118 mV/dec to 59–118 mV/dec.^69^ To sum up, none of the four ZnOHF-based electrocatalysts exhibited significant kinetic limitations for CO_2_RR. NW_75_–ZnOHF and NW_40_-ZnOHF showed enhanced HER hindering around the onset potential. This indicated that NW_75_–ZnOHF and NW_40_-ZnOHF achieve higher maximum FE values than P-ZnOHF and NW_20_-ZnOHF at lower overpotentials (Figure 2a).

At higher overpotentials (e.g., −1.0 V vs RHE), CO_2_RR is no longer governed solely by Tafel kinetics. The impact of intrinsic catalytic activity, shape factors, and surface area may dominate the CO_2_RR performance. Among these factors, electrochemical active surface area (ECSA) often plays a significant role in partial current densities of CO_2_RR products. To investigate whether ECSA serves as the principal factor governing the CO_2_RR performance of ZnOHF nanowire samples, double-layer capacitance (*C*_dl_) measurements were conducted to normalize the current densities. We performed CV scans for all the samples from 0.25 to 0.45 V vs Ag/AgCl at scan rates of 20, 40, 60, 80, and 100 mV/s (Figure S3c–f), and used the calculated *C*_dl_ to normalize the current densities for CO_2_RR and HER, as shown in Figures 3c,3d, and S3g. NW_40_-ZnOHF exhibited a markedly higher *j*_CO_ECSA_ than P-ZnOHF, NW_75_–ZnOHF, and NW_20_-ZnOHF at high overpotentials, implying that NW_40_-ZnOHF possessed greater intrinsic catalytic activity. In Figure 3d, *j*_H2_ECSA_ of NW_75_–ZnOHF was noticeably lower than that of the other three ZnOHF catalysts. This suppressed HER behavior is likely due to the high ECSA of NW_75_–ZnOHF, leading to the accumulation of surface OH^–^ during CO_2_RR or HER, thereby elevating the local pH and inhibiting HER.^43^ In contrast, HER was not notably suppressed in NW_20_-ZnOHF and NW_40_-ZnOHF at high overpotentials. Therefore, the greater intrinsic activity that worked with ECSA governed its CO_2_RR performance instead of HER hindering, resulting in a difference among all other samples in maximum FEs and corresponding CO partial current densities (Figure 2a,b).

### 3.4 Investigation of ZnO/ZnOHF Hybridized Heterointerfaces

To decouple the outperformed intrinsic activity for CO_2_RR of NW_40_-ZnOHF, which could be associated with the surface microstructure features such as crystal facets, fluorine atomic content, oxidation states, or the density of oxygen vacancies, etc.,^44–46^ multiple material analyses were conducted to investigate the microstructure of all the samples. XRD was employed to verify the compositions and crystal structures (Figure 4a). P-ZnOHF, NW_75_–ZnOHF, NW_40_-ZnOHF, and NW_20_-ZnOHF showed clear patterns of (110) at 20.6°, (310) at 32.4°, (111) at 35.4°, and (221) at 51.4°,^47^ corresponding to the standard ZnOHF sample. However, additional ZnO(002) facets was observed at 34.3° in all ZnOHF samples and caused a distorted short pattern next to the ZnOHF(111) main peak, more clearly in the nanowire samples especially for NW_40_-ZnOHF. For further investigation, TEM/EDS was conducted to observe NW_40_-ZnOHF. Figure 4b presented the TEM image and corresponding elemental EDS mappings of NW_40_-ZnOHF. From the EDS spectra (Figure S4a), NW_40_-ZnOHF was composed of 36.72% Zn, 16.51% F, and 46.78% O in atomic percentage. According to stoichiometry, the element ratios between Zn and F are 1:1 in standard ZnOHF. Note that we ignore the O atomic ratio since rich oxygen-containing organic contaminants exist in the samples. However, the Zn atomic ratio was twice the amount more than that of F, indicating the extra presence of Zn-based materials that did not contain fluorine in NW_40_-ZnOHF. Combining this observation with the XRD patterns, we inferred that ZnO (002) did exist and contributed to the increased Zn content in NW_40_-ZnOHF.

**Figure 4:**
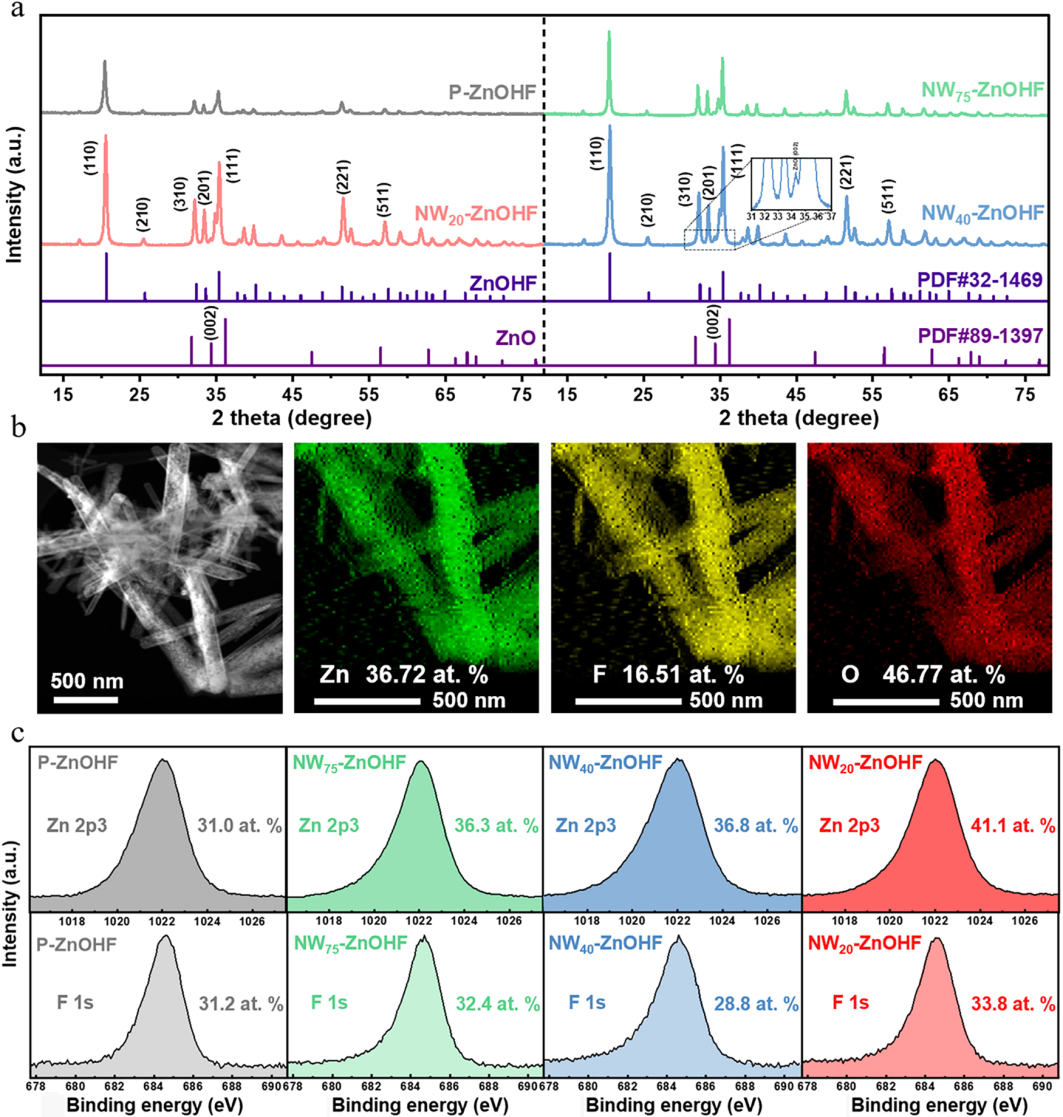
(a) XRD patterns of P-ZnOHF, NW_75_–ZnOHF, NW_40_-ZnOHF, and NW_20_-ZnOHF. (b) TEM image and elemental mapping of NW_40_-ZnOHF. (c) Zn 2p3 and F 1s XPS spectra of P-ZnOHF, NW_75_–ZnOHF, NW_40_-ZnOHF, and NW_20_-ZnOHF.

XPS was further implemented to investigate the “surface” elemental composition of all the samples. Note that the analytical depth of EDS and XRD exceeds 1 μm, whereas XPS is less than 10 nm. Figure S4b–e showed the XPS full spectra of P-ZnOHF, NW_75_–ZnOHF, NW_40_-ZnOHF, and NW_20_-ZnOHF. In each spectrum, the Zn 2p3 peak appeared at 1022 eV, Zn 2p1 at 1045 eV, F 1s at 684 eV, and O 1s at 531 eV. The respective surface atomic concentration for all the samples was presented in Figure 4c, obtained by calculating the peak areas, divided by the atomic sensitivity factor, from high-resolution scans for Zn 2p3, F 1s, and O 1s. The stoichiometric value for the surface Zn/F concentration ratio in P-ZnOHF was 0.99. Notably, the surface Zn concentration of NW_40_-ZnOHF is close to the surface F concentration (Zn/F = 1.27:1), relatively matching ZnOHF stoichiometry, which was a big contrast to the ratio (Zn/F = 2.22:1) measured by EDS but still showing the minor ZnO species in the surface (Figure S4a). The high-resolution TEM images provide evidence of the predominant ZnOHF facets (Figure S4f).

For further explanation, the slight difference in Zn: F surface concentration ratios among P-ZnOHF and other nanowires (Figure 4c) stemmed from the HMT-mediated formation of ZnO/ZnOHF heterointerfaces. HRTEM analysis (Figure 5a) indicated the presence of ZnO and ZnOHF domains of NW_40_-ZnOHF. Identification of the ZnO domain was based on the FFT pattern (Figure S5a,b). In Figure S5a, a ZnO(002) reflection analogous to the XRD results was observed, which does not correspond to any facet of ZnOHF. Likewise, the same feature was discerned in another HRTEM image of NW_40_-ZnOHF and corresponding FFT pattern (Figure S6a–c), thereby providing additional confirmation of the ZnO/ZnOHF hybridized structure. Greene et al. reported that ZnO nanowires can be synthesized from zinc salts and HMT, leading to ZnO growth along the [0001] direction of the stable wurtzite structure.^49^ This orientation typically gives rise to an intense (002) facet diffraction pattern in XRD.^50^ Based on the above evidence, the growth formation of the HMT-mediated ZnOHF nanowire could be described below: The abundant F^–^ ions provided by ZnF_2_ preferentially drive Zn^2+^ ions to react with F^–^ and OH^–^, forming ZnOHF, while a small portion of Zn^2+^ competed to react with OH^–^ to form Zn­(OH)_2_,^29^ resulting in minor incorporation of Zn­(OH)_2_ on the ZnOHF surface. In this process, Zn­(OH)_2_ underwent dehydration to ZnO.^48^ This ZnO crystal transformation was spatially restricted to specific localized regions on the ZnOHF nanowires, thereby creating a mixed ZnO/ZnOHF hybridized heterointerface.

**Figure 5:**
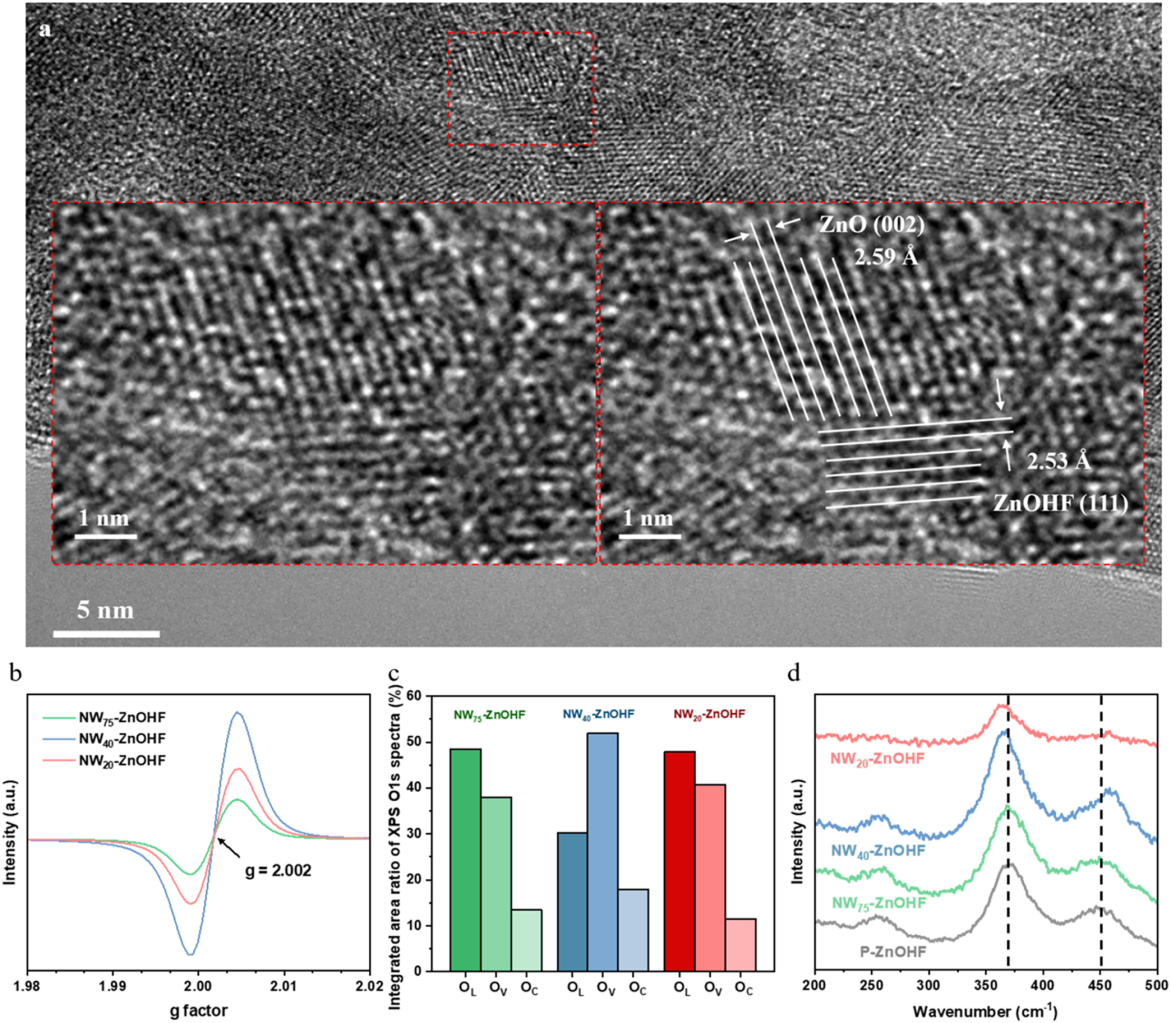
(a) HRTEM image of NW_40_-ZnOHF. (b) ECSA normalized EPR spectra of NW_75_–ZnOHF, NW_40_-ZnOHF, and NW_20_-ZnOHF. (c) Relative ratios of various oxygen species in NW_75_–ZnOHF, NW_40_-ZnOHF, and NW_20_-ZnOHF, determined by high-resolution XPS O 1s. (d) Raman spectra of P-ZnOHF, NW_75_–ZnOHF, NW_40_-ZnOHF, and NW_20_-ZnOHF.

EPR spectra of NW_75_–ZnOHF, NW_40_-ZnOHF, and NW_20_-ZnOHF (Figure S7a) confirm the presence of paramagnetic centers associated with oxygen vacancies. All three samples exhibit a pronounced resonance at *g* ≈ 2.002, characteristic of electrons trapped at oxygen-vacancy sites.^64^ A qualitative comparison of the peak-to-peak signal intensities indicates that thicker nanowires show weaker signals, implying a lower concentration of oxygen vacancies. To evaluate whether these vacancies contribute to the intrinsic CO_2_RR activity, each EPR spectrum was normalized by the ECSA of the corresponding sample (Figure 5b). Notably, NW_40_-ZnOHF displayed the highest oxygen vacancy density per unit surface area. The existence of surface oxygen vacancies could be also verified by high-resolution XPS of O 1s spectrum with NW_75_–ZnOHF, NW_40_-ZnOHF, and NW_20_-ZnOHF (Figure S7b–d). O 1s peak was deconvoluted into lattice oxygen in Zn–O (O_L_, 530 eV), oxygen vacancies (O_V_, 531.2 eV), and adsorbed oxygen (O_C_, 532.2 eV).^53–55^ The respective fractions of oxygen species were summarized in Figure 5c. NW_40_-ZnOHF exhibited a noticeably higher surface concentration of O_V_ than NW_75_–ZnOHF and NW_20_-ZnOHF. This was consistent with the observation of EPR results. Low-frequency Raman spectroscopy was employed to analyze all the samples, as shown in Figures 5d and S8a–d. All the samples exhibits the polar Zn–O/F out-of-phase stretching mode A_2u_ along the *c*-axis near 450 cm^–1^, as well as the Zn–O/F out-of-phase stretching mode E_u_ in the a- and *b*-axis near 369 cm^–1^.^51^ Obviously, the A_2u_ and E_u_ modes of nanowire samples shifted in the opposite direction compared to the P-ZnOHF, which might result from octahedral distortion or an uneven distribution of OH^–^/F^–^ in the ZnOHF structure.^51^ The uneven OH^–^/F^–^ distributions were highly likely attributed to the oxygen vacancies.^52^ The oxygen vacancies are thought to originate during the hydrothermal synthesis step.^56^

### 3.5 Structure Reconstruction by CO_2_RR

Electrochemical reconstruction is an unavoidable phenomenon during CO_2_RR and can appreciably influence catalytic performance.^43^ Accordingly, we examined the structural evolution of ZnOHF nanowires after 20 min electrolysis at −1.0 V vs RHE. GIXRD patterns collected post 20 min CO_2_RR are shown in Figure S9a. Reflections at 18° and 26.5° originate from the carbon-based gas-diffusion electrode (GDE) (reference pattern in Figure S9a), whereas the peak at 20.7° is assigned to the (100) facet of ZnOHF. A magnified view of 2θ = 28.0–43.6° (Figure 6a–6c) reveals that the ZnOHF (310), (201), and (111) reflections persist in all three samples, indicating that the bulk ZnOHF lattice remains during short-term CO_2_RR. By contrast, the ZnO (002) reflection vanishes in NW_20_-ZnOHF and NW_75_–ZnOHF but remains clearly visible in NW_40_-ZnOHF, implying that the ZnO/ZnOHF hybridized heterointerface in NW_40_-ZnOHF is more resistant to cathodic reconstruction. However, trace metallic Zn(100) and ZnF_2_(200) patterns emerge, evidencing the unavoidable structure reconstruction under CO_2_RR conditions.

**Figure 6:**
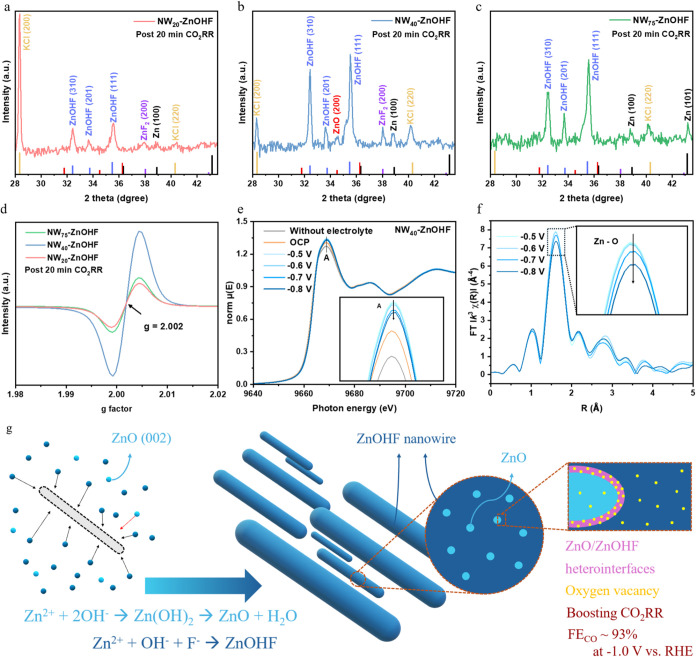
Enlarged GIXRD patterns after 20 min CO_2_RR for (a) NW_20_-ZnOHF, (b) NW_40_-ZnOHF, and (c) NW_75_–ZnOHF in the 2θ range of 28–43.6°. (d) C_dl_ normalized EPR spectra after 20 min CO_2_RR for NW_75_–ZnOHF, NW_40_-ZnOHF, and NW_20_-ZnOHF. (e) in situ XAS and (f) FT-EXAFS spectra for NW_40_-ZnOHF. (g) A schematic illustration of ZnOHF based nanowires with enriched oxygen vacancies in the ZnO/ZnOHF hybridized heterointerfaces for achieving high CO_2_RR activity.

Comparative EPR spectra acquired before and after 20 min of CO_2_RR at −1.0 V vs RHE are presented in Figure S9b. The *g* ≈ 2.002 resonance of NW_20_-ZnOHF diminishes markedly after CO_2_RR, indicating a depletion of oxygen vacancies, whereas NW_40_-ZnOHF and NW_75_–ZnOHF show increases in signal intensity. For comprehensively compared to the pristine EPR results, the post CO_2_RR EPR spectra was then normalized by the ECSA (Figure 6d). After normalization, the post 20 min CO_2_RR NW_40_-ZnOHF displays the highest oxygen-vacancy density per unit surface area, so does its pristine state. Post-CO_2_RR XPS O 1s spectra (Figure S9c) corroborate the EPR findings, revealing that NW_40_-ZnOHF retains a substantially higher concentration of oxygen vacancies. Despite NW_40_-ZnOHF undergoing unavoidable electrochemical reconstruction but with a sustained ZnO/ZnOHF hybridized heterointerface, it not only preserve but also generate a favorable concentration of oxygen vacancies during CO_2_RR.^65,66^ The enriched surface O_V_ was regarded as the critical facilitator for enhancing the intrinsic CO_2_RR activity since their capability to lower the free energy of CO_2_RR intermediates (COOH*) reported by many studies.^24,57^

Great interest was aroused since the increased O_v_ concentration of NW_40_-ZnOHF was confirmed by post CO_2_RR EPR. Therefore, in situ XAS was further employed to monitor the dynamic structural evolution in more detail of NW_40_-ZnOHF during CO_2_RR (Figure 6e,6f). In Figure 6e, the white line recorded between −0.5 and −0.8 V vs RHE is essentially identical to that at open-circuit potential (OCP) or in the electrolyte-free state, indicating that NW_40_-ZnOHF does not undergo noticeable structure evolution within this potential window. The intensity of feature A is markedly higher at OCP than in the absence of electrolyte, suggesting that electrolyte introduction promptly gives rise to chemisorption on the NW_40_-ZnOHF surface and polarizes the Zn electron toward the adsorbate. Notably, when −0.5 V vs RHE is applied, the additional formation of Zn–CO_2_^ads^ further enhances. However, upon stepping to more negative potentials, Zn–CO_2_^ads^ undergoes CO_2_RR to CO and subsequently desorbs, causing a slight decrease in the amplitude. Figure 6f presents the Fourier-transformed EXAFS (FT-EXAFS) spectra of NW_40_-ZnOHF collected under identical conditions. The gradual suppression of the Zn–O peak and the slightly shift toward larger radius with increasingly negative potentials likely reflects lattice distortion in NW_40_-ZnOHF induced by the ongoing CO_2_RR.^67^ This might cause an amorphous surface and further the formation of oxygen vacancies.^66,68^ Overall, in situ XAS evidence the limited structural evolution of NW_40_-ZnOHF, matching post GIXRD results, while unfortunately it could not provide direct evidence of the existence of O_v_ and the newly formation of O_v_ during CO_2_RR. However, based on the strong evidence of EPR before and after CO_2_RR, we can still reasonably infer that highest FE in NW_40_-ZnOHF attributed to the high-level oxygen vacancy preexisted in the pristine structure and newly formed oxygen vacancies by CO_2_RR evolution (Schematic Figure 6g). This critically influences intrinsic catalytic performance.

### 3.6 Long-Term Electrocatalytic Stability

To examine stability as an electrocatalyst, NW_40_-ZnOHF experienced a 12 h CO_2_RR test at −1.0 V versus RHE to evaluate its longer-term stability. As shown in Figure 7a, the current density stayed between −18 to −21 mA/cm^2^ throughout the 12 h CO_2_RR, and FE_CO_ remained above 90% for the first 9 h yet gradually decreased to 85.6% by 12 h. These results suggest that NW_40_-ZnOHF maintains much of its activity during prolonged CO_2_RR operation, indicating a decent level of durability for CO production.

**Figure 7:**
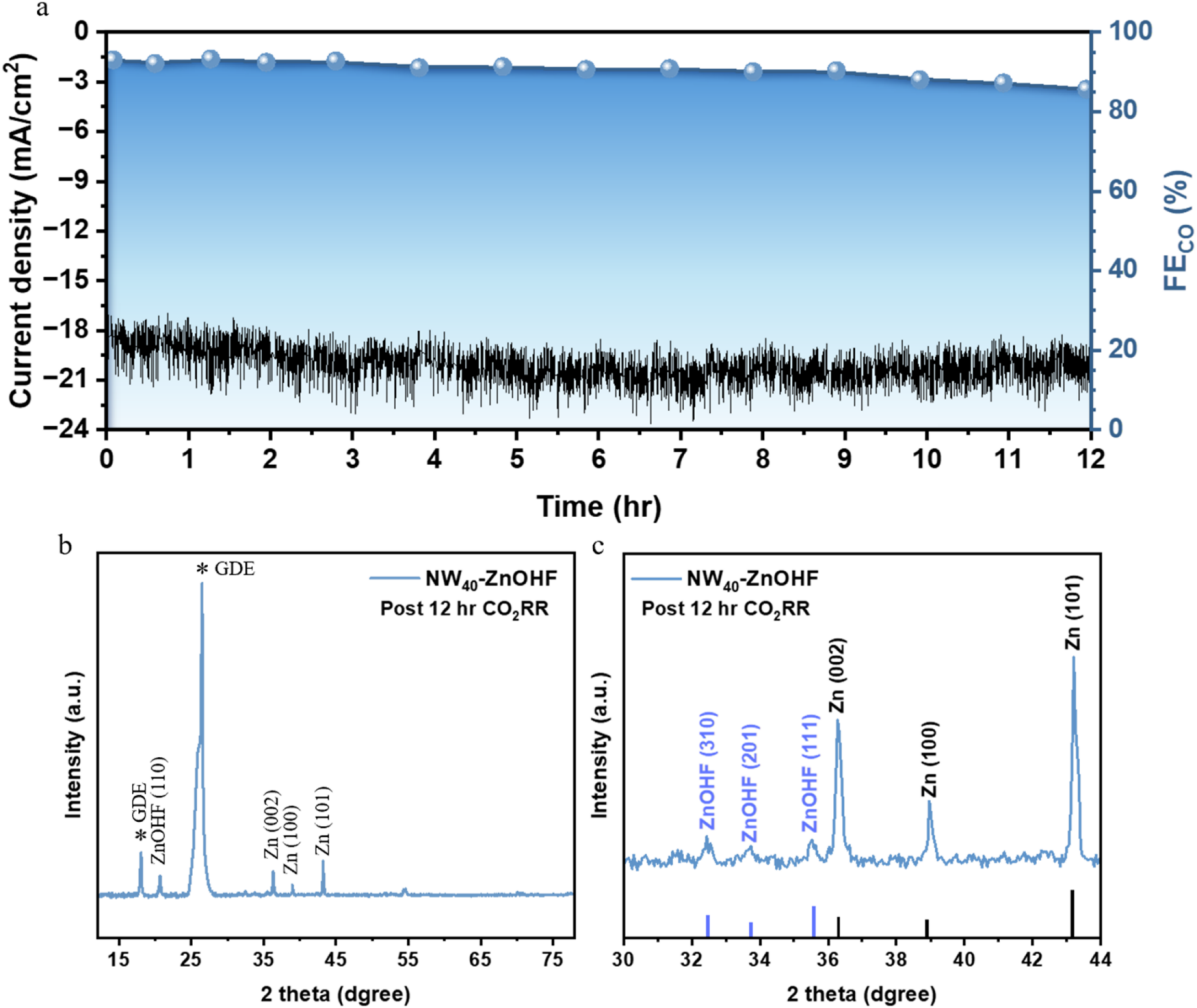
(a) 12 h CO_2_RR stability test of NW_40_-ZnOHF at −1.0 V vs RHE. Post 12 h CO_2_RR (b) GIXRD pattern and (c) enlarged in the 2θ range of 30–44°.

Given that a 12 h continuous CO_2_RR operation could induce more severe electrochemical reconstruction of NW_40_-ZnOHF, we conducted post-CO_2_RR structural analyses. The post 12 h CO_2_RR GIXRD pattern shows that the ZnOHF(100) reflection at 2θ = 20.7° is markedly attenuated compared with the sample tested for 20 min post CO_2_RR (Figure 7b). In contrast, the metallic Zn reflections at 36.3, 39.0, and 43.3°, corresponding to the (002), (100), and (101) planes, respectively, become more pronounced. The magnified region of the 2θ = 30–44° region (Figure 7c) shows weaken residual signals of ZnOHF, and the ZnO(002) reflection disappears entirely. These observations suggest that, as CO_2_RR proceeds over long durations, the ZnO/ZnOHF heterointerface gradually vanishes. This finding indicates that the intrinsic CO_2_RR activity originally imparted by the enriched oxygen vacancies existing in the ZnO/ZnOHF hybridized heterointerfaces fade and the structure reconstruction replaced by metallic Zn with prolonged operation, leading to the decline in FE_CO_.

## 4 Conclusions

This work successfully synthesized different scales of ultrathin ZnOHF based nanowires with ZnOHF/ZnO hybridized heterointerfaces and unveiled the key facilitator for enhancing the intrinsic CO_2_RR activity associated with the enriched oxygen vacancies concentrations. NW_40_-ZnOHF exhibited the highest CO_2_RR selectivity in an H-cell, achieving an FE of 93% and a *j*_CO_ of −17.2 mA cm^–2^ at −1.0 V vs RHE. NW_40_-ZnOHF displayed decent performance of over FE 90% for 9 h in a stability test without significant structure reconstruction. This finding highlights the significance of oxygen vacancies created during heterointerface formation for guiding the rational design of transitional metal-based electrocatalysts for use in advanced CO_2_RR technology.

## Supplementary Material



## Notes

Additional figures for morphology, structure, and surface chemistry of P-ZnOHF and NW_*x*_-ZnOHF including SEM, TEM/EDS, HRTEM/FFT, XPS (survey and O 1*s*), EPR, and low-frequency Raman; electrochemical data in H-cells (FE_H2_, *j*_total_, j_H2_, ECSA-corrected jH2, LSV in N_2_ and CO_2_, and CV scan); post-CO_2_RR (20 min) analyses (XRD, EPR, XPS) (PDF)
