# Supplementary material for: Ultrathin Hybridized ZnOHF Nanowires with Enriched Oxygen Vacancies for High Selective CO2‑to-CO Electrocatalytic Conversion
Source: ACS Appl Mater Interfaces. 2025 Sep 8;17(37):52064–76. doi: 10.1021/acsami.5c10915 (PMC12447396; doi:10.1021/acsami.5c10915)
Supplement: Supplementary file 1 [file am5c10915_si_001.pdf]

Supporting Information

Ultrathin Hybridized ZnOHF Nanowires with Enriched Oxygen Vacancies for High Selective CO_2-to-CO Electrocatalytic Conversion

Hsin-Chiao Wu†[1], Chia-Chen Lin†[1], Yu-Jen Chou‡, Shu-Yu Lee†‡, Shih-Han Wang†‡, Tsan-Yao Chen†‡, Ta-Chung Liu†*

† Department of Biomedical Engineering, National Yang Ming Chiao Tung University, Taipei 112, Taiwan.

‡ Department of Mechanical Engineering, National Taiwan University of Science and Technology, Taipei 106, Taiwan.

†‡Department of Engineering and System Science, National Tsing Hua University, Hsinchu 300, Taiwan.

1 These authors contributed equally: Hsin-Chiao Wu, Chia-Chen Lin

*Corresponding author's e-mail: tcliu@nycu.edu.tw

Figure S1. SEM image of P-ZnOHF.

Figure S2. The (a) FE_{H_2}, (b) j_{total}, and (c) j_{H_2} plots of P-ZnOHF, NW_{75}-ZnOHF, NW_{40}-ZnOHF, and NW_{20}-ZnOHF in H-cells.

Figure S3. LSV scan of P-ZnOHF, NW_{75}-ZnOHF, NW_{40}-ZnOHF, and NW_{20}-ZnOHF in (a) N_2- and (b) CO_2-satrated conditions. CV scan of (c) P-ZnOHF, (d) NW_{75}-ZnOHF, (e) NW_{40}-ZnOHF, and (f) NW_{20}-ZnOHF. (g) The ECSA corrected j_{H_2} of P-ZnOHF, NW_{75}-ZnOHF, NW_{40}-ZnOHF, and NW_{20}-ZnOHF.

Figure S4. (a) TEM/EDS spectra of NW_{40}-ZnOHF. XPS spectra of (b) P-ZnOHF, (c) NW_{75}-ZnOHF, (d) NW_{40}-ZnOHF, and (e) NW_{20}-ZnOHF. (f) High resolution TEM image of NW_{40}-ZnOHF and FFT image of corresponding regions.

Figure S5. (a) Fast Fourier transform (FFT) of **Figure 5a** in the main text. (b) The d-spacings corresponding to ZnOHF and ZnO XRD peaks in the 2θ range of 32–36° are highlighted with black rings.

Figure S6. HRTEM of NW_{40}-ZnOHF and corresponding FFT pattern.

Figure S7. (a) EPR spectra of NW_x-ZnOHF electrodes. The O1s XPS spectra of (b) NW_{75}-ZnOHF, (c) NW_{40}-ZnOHF, and (d) NW_{20}-ZnOHF.

Figure S8. Low frequency Raman spectra of (a) P-ZnOHF, (b) NW_{75}-ZnOHF, (c) NW_{40}-ZnOHF, and (d) NW_{20}-ZnOHF.

Figure S9. Post 20 min CO_2RR (a) XRD pattern, (b) EPR spectra, and (c) O1s XPS spectra for NW_x-ZnOHF.

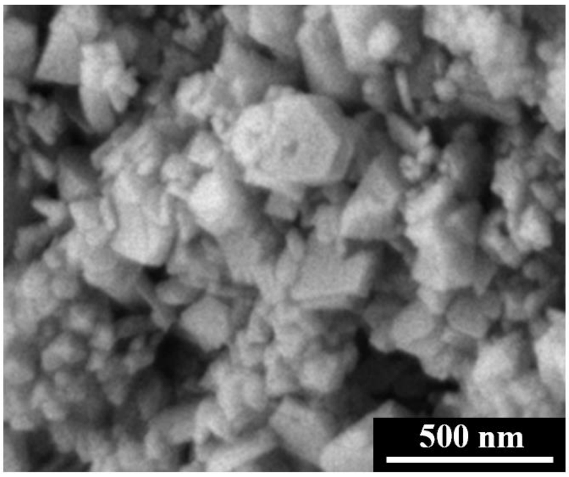

Figure S1. SEM image of P-ZnOHF.

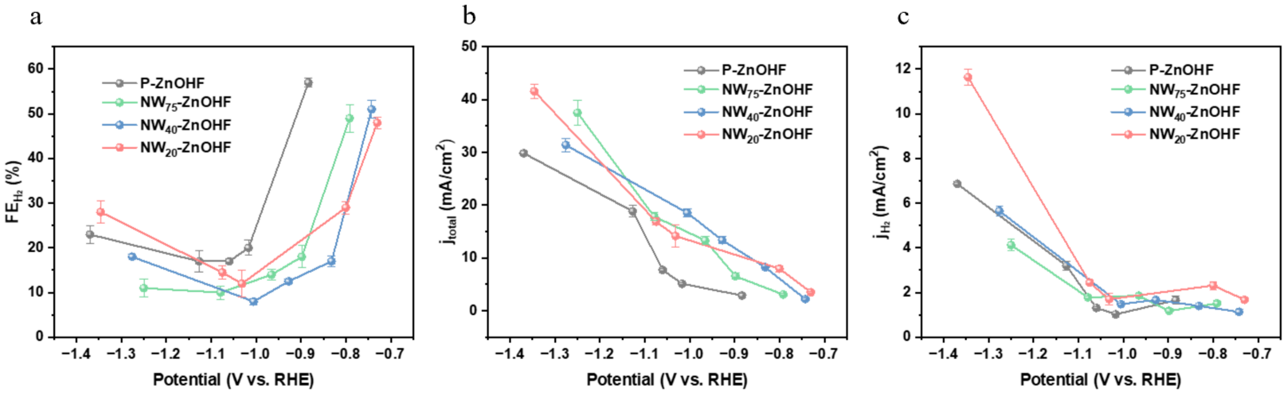

Figure S2. The (a) FE_{H2}, (b) j_{total}, and (c) j_{H2} plots of P-ZnOHF, NW_{75}-ZnOHF, NW_{40}-ZnOHF, and NW_{20}-ZnOHF in H-cells.

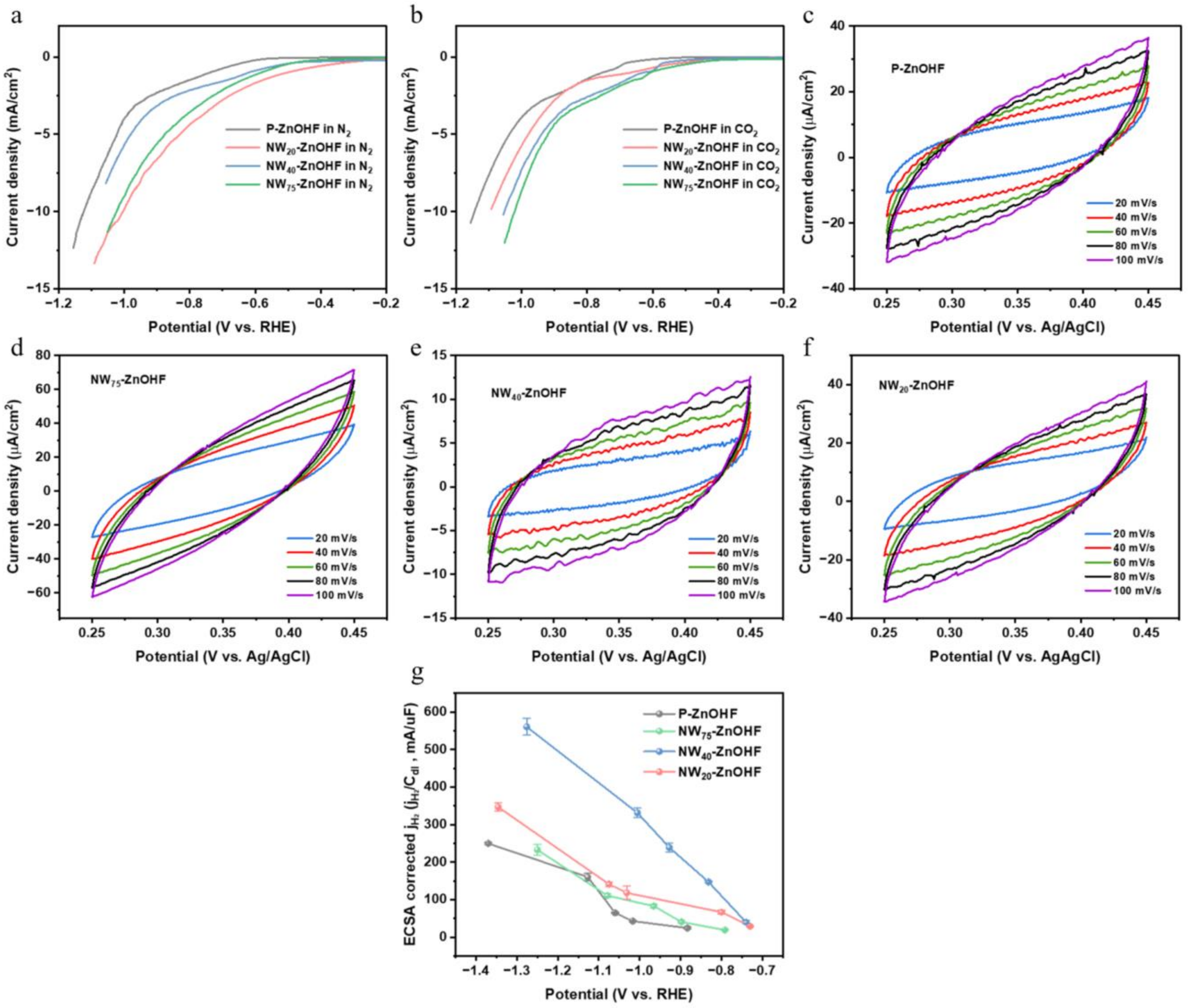

Figure S3. LSV scan of P-ZnOHF, NW_{75}-ZnOHF, NW_{40}-ZnOHF, and NW_{20}-ZnOHF in (a) N_2- and (b) CO_2-satrated conditions. CV scan of (c) P-ZnOHF, (d) NW_{75}-ZnOHF, (e) NW_{40}-ZnOHF, and (f) NW_{20}-ZnOHF. (g) The ECSA corrected j_{H2} of P-ZnOHF, NW_{75}-ZnOHF, NW_{40}-ZnOHF, and NW_{20}-ZnOHF.

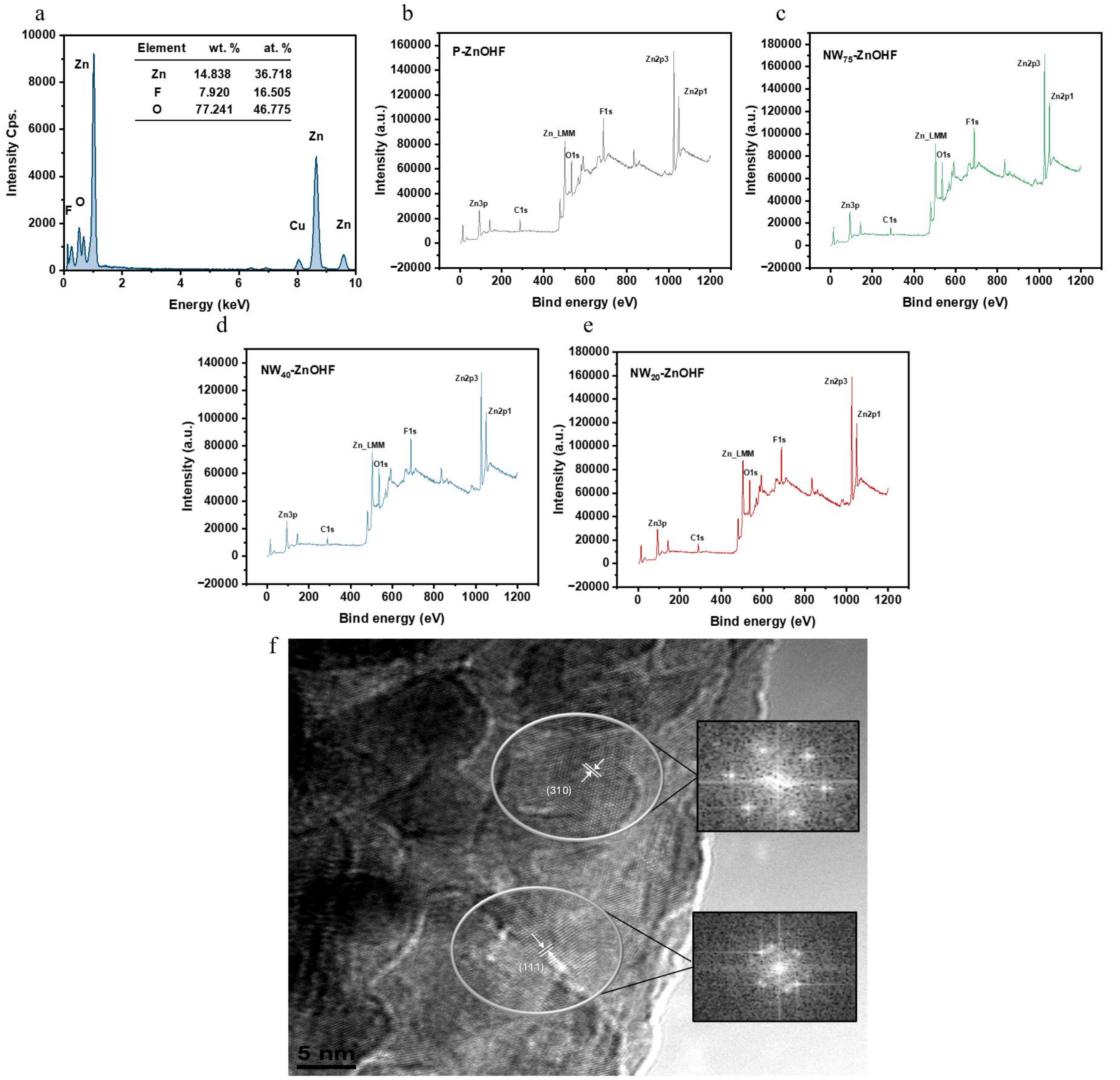

Figure S4. (a) TEM/EDS spectra of NW_{40}-ZnOHF. XPS spectra of (b) P-ZnOHF, (c) NW_{75}-ZnOHF, (d) NW_{40}-ZnOHF, and (e) NW_{20}-ZnOHF. (f) High resolution TEM image of NW_{40}-ZnOHF and FFT image of corresponding regions.

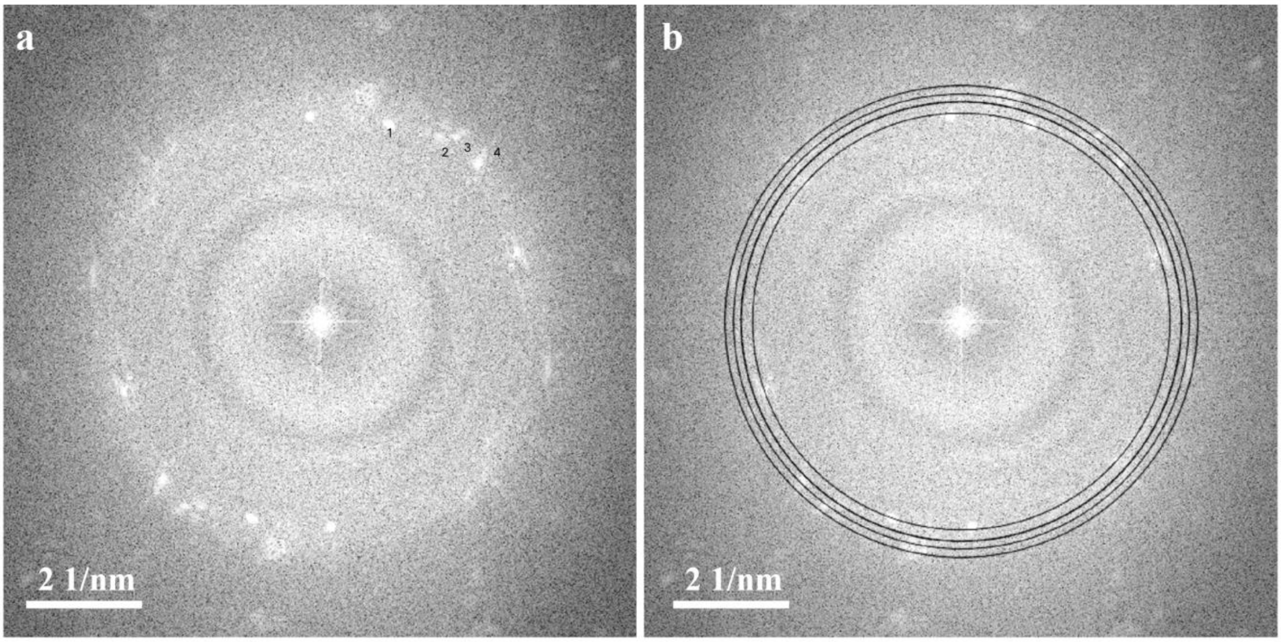

Figure S5. (a) Fast Fourier transform (FFT) of **Figure 5a** in the main text. (b) The d-spacings corresponding to ZnOHF and ZnO XRD peaks in the 2θ range of 32–36° are highlighted with black rings.

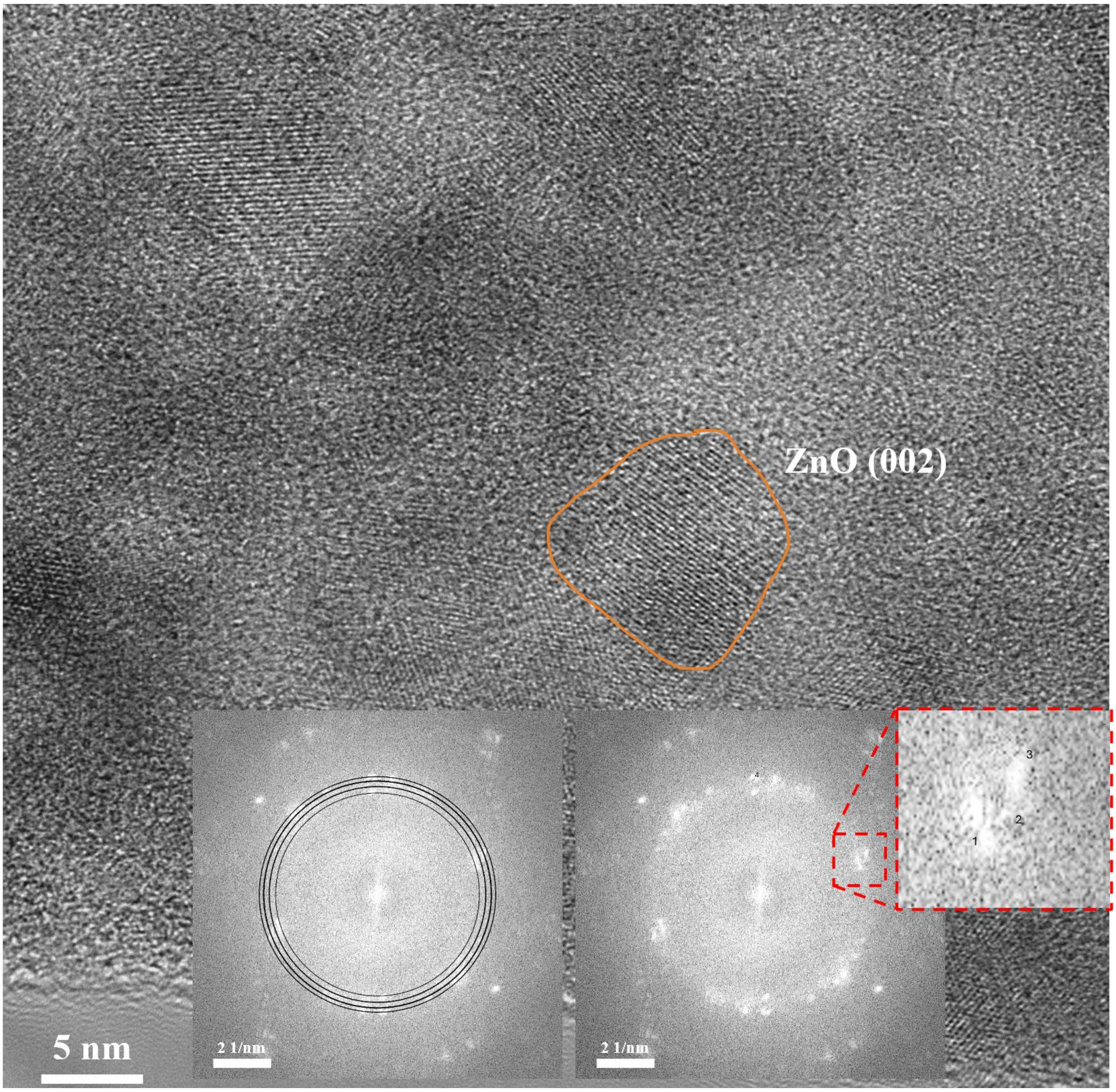

Figure S6. HRTEM of NW_{40}-ZnOHF and corresponding FFT pattern.

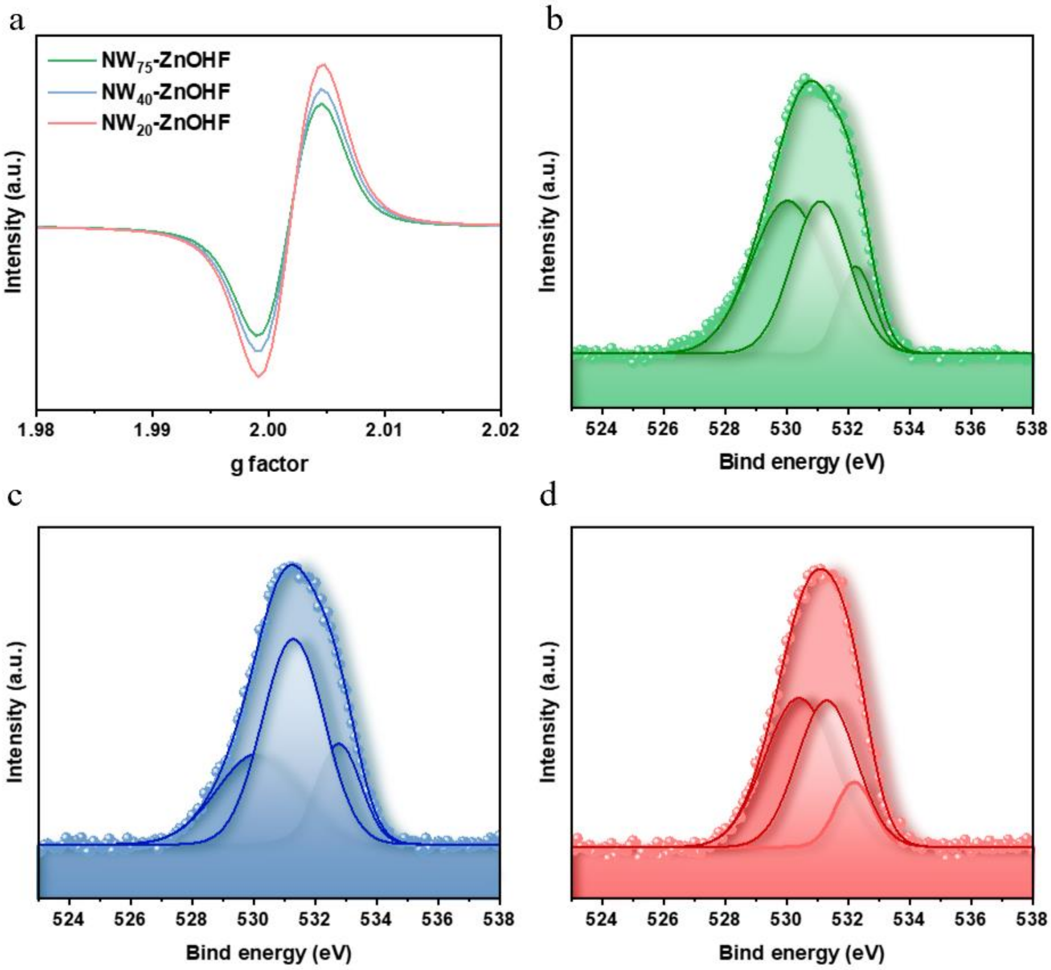

Figure S7. (a) EPR spectra of NW_x-ZnOHF electrodes. The O1s XPS spectra of (b) NW_{75}-ZnOHF, (c) NW_{40}-ZnOHF, and (d) NW_{20}-ZnOHF.

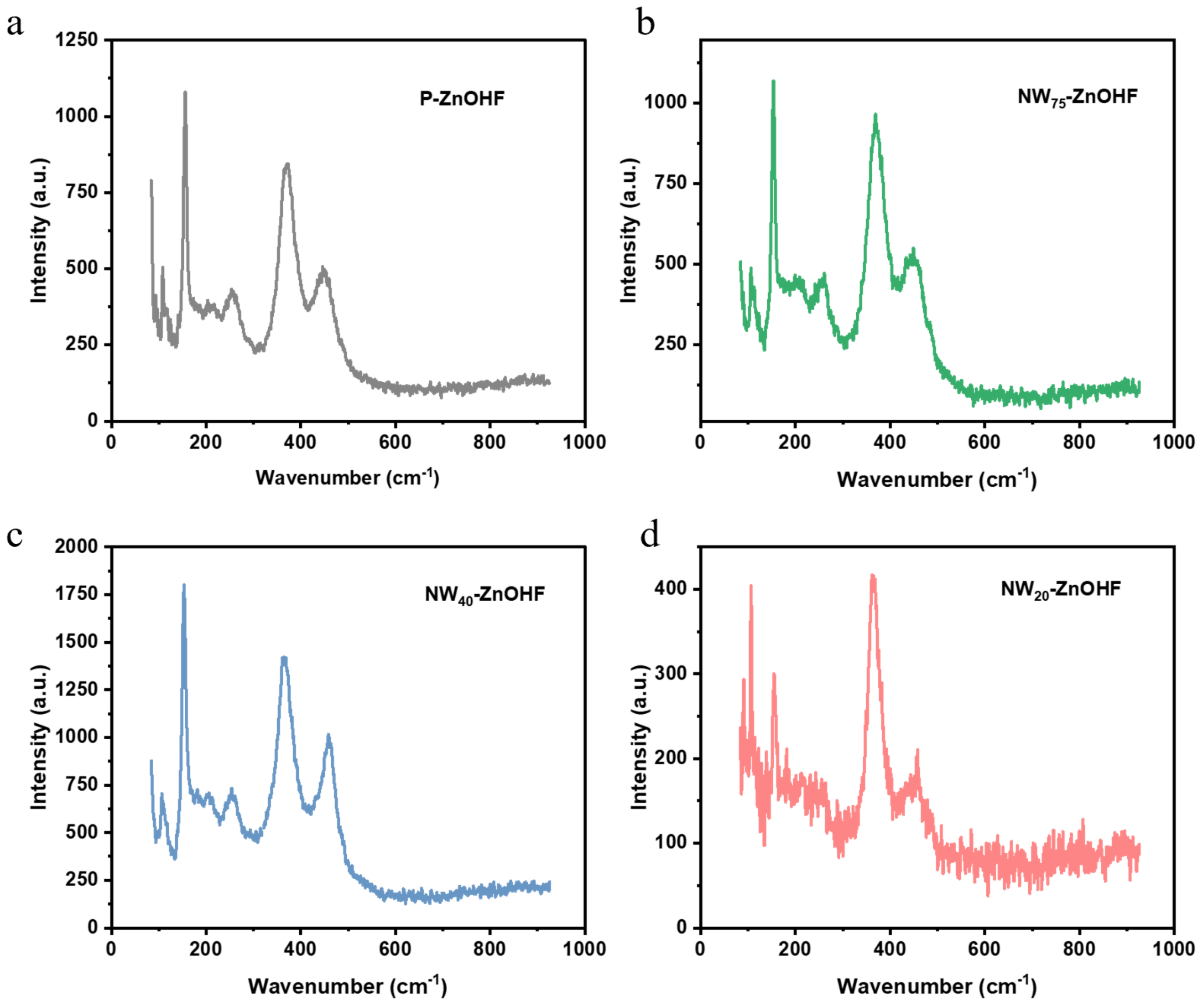

Figure S8. Low frequency Raman spectra of (a) P-ZnOHF, (b) NW_{75}-ZnOHF, (c) NW_{40}-ZnOHF, and (d) NW_{20}-ZnOHF.

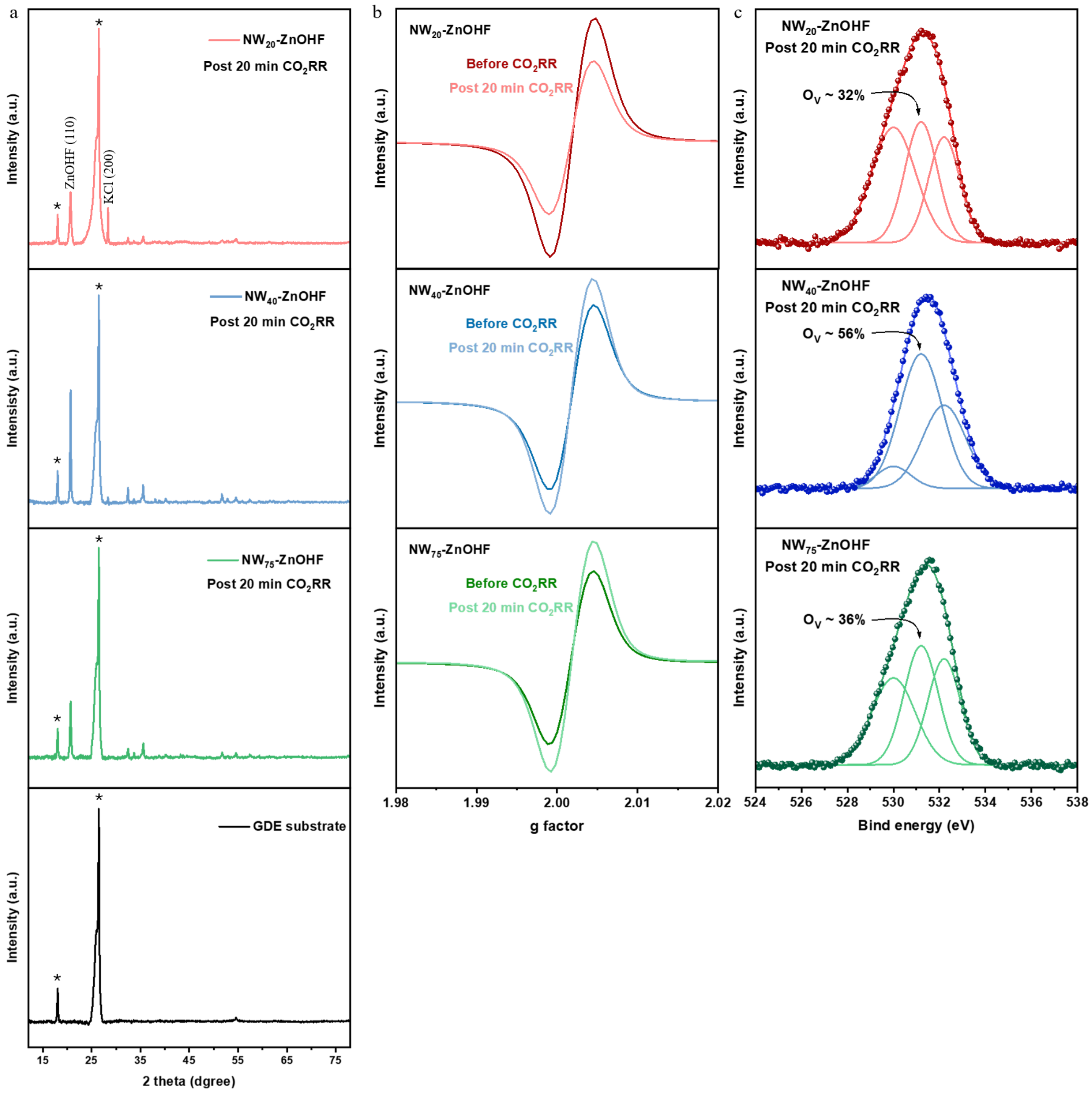

Figure S9. Post 20-min CO_2RR (a) XRD pattern, (b) EPR spectra, and (c) O1s XPS spectra for ZnOHF nanowire samples.
